# May-Thurner Syndrome, a Frequently Overlooked Cause of Iliofemoral Vein Thrombosis

**DOI:** 10.7759/cureus.80212

**Published:** 2025-03-07

**Authors:** David Sabat, Alyson Skelly, Alejandro Biglione

**Affiliations:** 1 Dr. Kiran C. Patel College of Osteopathic Medicine, Nova Southeastern University, Fort Lauderdale, USA; 2 Internal Medicine, Wellington Regional Medical Center, Wellington, USA

**Keywords:** deep vein thrombosis (dvt), iliac vein stent, illiac vein compression, may-thurner's syndrome, mechanical thrombectomy (mt), venous anomaly

## Abstract

May-Thurner syndrome (MTS) involves the compression of the left iliofemoral vein by the right common iliac artery against the vertebral body, creating an environment conducive to venous stasis and endothelial injury, which predisposes patients to deep vein thrombosis (DVT). Although often asymptomatic, MTS can present with lower extremity swelling and claudication. We present a case of a 72-year-old female who developed extensive left lower extremity DVT. Imaging studies, including Doppler ultrasound and venography, confirmed the diagnosis of MTS with significant vessel occlusion. Treatment involved mechanical thrombectomy, angioplasty, and iliac vein stenting. The patient demonstrated rapid symptom resolution post-procedure and was discharged on anticoagulation therapy. This case underscores the importance of recognizing anatomical factors like MTS in patients with DVT and highlights the role of interventional management in preventing complications.

## Introduction

Deep vein thrombosis (DVT) occurs when blood clots form within the deep venous system and typically occurs in the large veins of the legs or pelvis. In the general population, the incidence of DVT is estimated to be 67 per 100,000 per year [1]. First proposed by Rudolf Virchow, the primary factors contributing to the formation of blood clots include venous stasis, blood hypercoagulability, and vascular wall injury [2]. Patients commonly present with unilateral leg pain, redness, and swelling [2]. Since DVTs can dislodge, travel through the heart, and embolize in the pulmonary arteries, it is essential to diagnose and manage patients accurately and quickly.

May-Thurner syndrome (MTS) is an anatomical condition in which the left iliofemoral vein is compressed against the vertebral body by the right common iliac artery [3]. Initially documented in 1957, this vascular anomaly was characterized by persistent pulsations of the right common iliac artery, which overlies the left common iliac vein. Over time, this results in the deposition of elastin and collagen eventually causing intimal fibrosis along the vein wall [3]. These alterations can eventually lead to the narrowing of the vessel lumen, resulting in venous flow irregularities, venous hypertension, and possibly DVT [3]. Given that most patients are asymptomatic, the true prevalence is unknown; however, in an asymptomatic population evaluated by computed tomography, the prevalence was found to be 24% [4]. In the setting of DVT, the prevalence can range from 18-49% [5].

This paper presents a rare case of MTS leading to extensive DVT and discusses its diagnosis and management.

## Case presentation

A 72-year-old female with a history of peripheral vascular disease, hyperlipidemia, hypertension, heart failure with preserved ejection fraction (HFpEF), chronic obstructive pulmonary disease (COPD), and hypothyroidism was referred to the emergency department by her orthopedist due to complaints of left lower extremity pain and swelling. Her surgical history includes left shoulder arthroplasty, two cesarean sections, and revascularization of bilateral lower extremities with stenting. Notably, she underwent intramedullary fixation of an intertrochanteric femur fracture four weeks prior to presentation. After surgery, she was discharged to a rehabilitation facility with enoxaparin 40 mg/kg once daily for thromboprophylaxis. However, she left against medical advice after one week and did not comply with her thromboprophylactic regimen, increasing her risk for thromboembolic complications. She has no personal or family history of venous thromboembolism (VTE), deep vein thrombosis (DVT), or pulmonary embolism (PE). Daily medications include amlodipine 10 mg, aspirin 81 mg, atenolol 25 mg, atorvastatin 10 mg, and levothyroxine 75 mcg.

During her assessment in the emergency department, her vital signs were stable: she had a normal temperature of 36.8 °C, a heart rate of 78 beats/minute, and a respiratory rate of 17 breaths/min. However, she presented with hypertension, showing a blood pressure of 172/77 mmHg. Pulse oximetry revealed 96% saturation on room air. Notably, her left lower extremity displayed significant swelling and erythema in comparison to the right side. Lungs were clear to auscultation bilaterally, and her heart rhythm was regular with no murmurs detected. There were no signs of jugular venous distention, and her abdomen was soft, non-distended, and non-tender, with normal bowel sounds.

**Figure 1:**
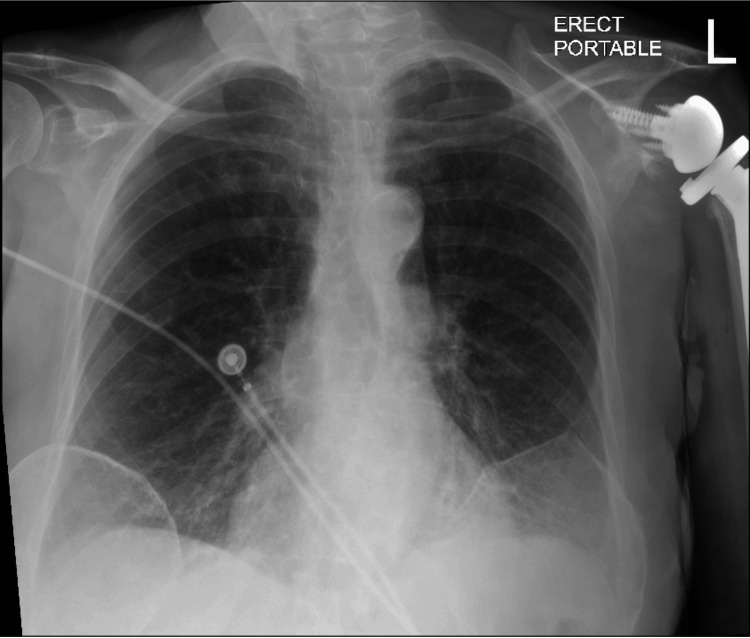
Patient's X-ray showing pulmonary vascular congestion, borderline cardiomegaly, and previous left shoulder arthroplasty

Initial laboratory results (Table 1) revealed normal coagulation parameters (PT: 10.1 seconds, PTT: 27.6 seconds, INR: 0.99), mild hypokalemia (potassium: 3.4 mmol/L), and no evidence of anemia or leukocytosis. Pro-B-type natriuretic peptide (pro-BNP) levels were high, consistent with her past diagnosis of HFpEF. Chest X-ray showed pulmonary vascular congestion, borderline cardiomegaly, and previous left shoulder arthroplasty (Figure 1).

**Table 1:** A summary of initial laboratory values upon presentation to the emergency department PTT: Partial Thromboplastin Time; PT: Prothrombin Time; INR: International Normalized Ratio; Hgb: Hemoglobin; Pro-BPN: Pro-B-Type Natriuretic Peptide; BUN: Blood Urea Nitrogen

Laboratory test	Value	Reference Range	Units
PTT	27.6	24.0 - 35.0	seconds
PT	10.1	9.7 - 11.7	seconds
INR	0.99	0.90 - 1.20	-
Hgb	13.2	12.0 - 16.0	g/dL
Leukocytes	7.77	4.50 - 10.50	x 10^3^/uL
Pro BNP	3019	≤ 215	pg/mL
Sodium	137	135 - 148	mmol/L
Potassium	3.4	3.6 - 5.2	mmol/L
BUN	7	7-18	mg/dL
Creatinine	0.79	0.55 - 1.02	mg/dL
Troponin	9.4	0 - 53.7	ng/L

**Figure 2:**
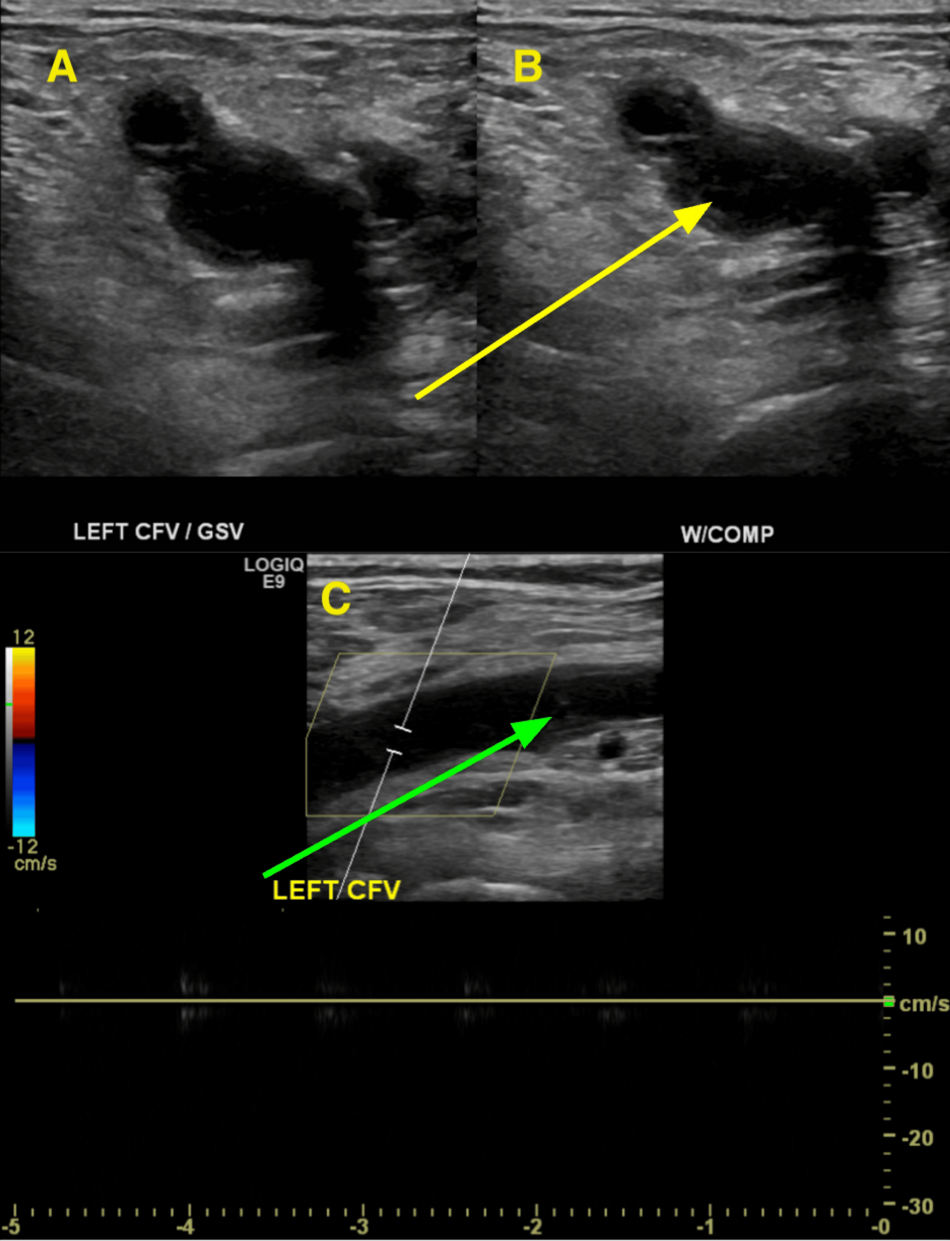
Sonography of the patient’s left common femoral vein (CFV) and great saphenous vein (GSV) in the transverse plane Image A shows the left CFV and GSV without compression with the ultrasound probe. Image B depicts the left CFV and GSV with compression with the ultrasound probe. Lack of vein compressibility (yellow arrow) indicated evidence of large thrombosis. Image C shows a longitudinal view of the left CFV with Doppler showing a total lack of blood flow. There is also evidence of echogenic thrombus (green arrow).

Doppler ultrasound (Figure 2) revealed extensive DVT from the left common femoral veins (CFV) down through the calf veins. The patient was initiated on a therapeutic dose of enoxaparin in the emergency room and admitted for further evaluation and treatment. Due to the extent of DVT, a vascular surgery consultation was obtained.

**Figure 3:**
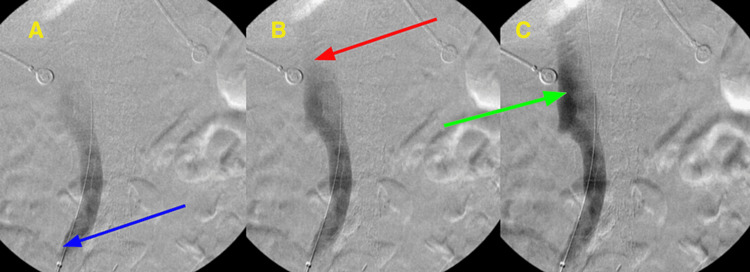
A sequence of images during venous fluoroscopy with contrast Image A shows the introduction of contrast via the tip of a catheter (blue arrow) into the left common iliac vein. Image B shows loss of continued flow at the point of compression (red arrow). Image C shows a pooling of contrast prior to the area of compression (green arrow).

An urgent venogram was performed due to the extent of thrombosis and the risk of massive pulmonary embolus. The venogram revealed extensive thrombosis in the left popliteal, femoral, common femoral, and iliac veins. An IVC filter was placed to prevent pulmonary embolism, and a mechanical thrombectomy was conducted. At the proximal common iliac vein, there was significant vessel occlusion, recoil of contrast, and disease consistent with MTS (Figure 3). Multiple angioplasties were performed, and 10 mm stents were deployed extending from the very proximal left common iliac vein into the common femoral vein. Post-stent placement venography showed adequate flow without residual stenosis or recoil of contrast.

One day after her procedure, her leg swelling significantly improved. She no longer had pain or erythema, and she denied shortness of breath, chest pain, or paresthesias in her lower extremities. She was discharged with postoperative management including oral anticoagulation with apixaban and antiplatelet therapy with clopidogrel for 30 days; after which she would resume daily aspirin.

## Discussion

MTS is an anatomical condition in which the left iliofemoral vein is compressed against the vertebral body by the right common iliac artery after it originates from the abdominal aorta and before the iliofemoral junction [3]. The compression causes decreased blood flow and increased turbulence in the left iliac vein, and over time, leads to venous stasis and endothelial injury. Therefore, patients with MTS are at increased risk of DVT formation. Although it most commonly presents on the left side, other compression scenarios include right-sided MTS and caval compression [6]. It classically presents as lower extremity swelling, claudication, hyperpigmentation, varicose veins, and/or venous ulceration [7].

The gold standard to diagnose a symptomatic MTS is intravascular ultrasound, however, computed tomography angiography, color Doppler ultrasound, and magnetic resonance imaging can also evaluate the iliac veins [8]. Management is guided by the presence or absence of thrombosis. Patients with thrombosis can be managed with thrombolysis, thrombectomy, stenting, and angioplasty, while symptomatic patients without thrombosis can be managed with stenting. Importantly, there is no indication to treat asymptomatic MTS [8].

This case report discussed the presentation of a 72-year-old female who, without a prior history of DVT, exhibited extensive thromboses in the left lower extremity. Notably, during thrombectomy, imaging revealed evidence of chronic disease resulting from compression of the common iliac vein, suggesting a previously asymptomatic MTS until presentation.

Several factors contribute to the complexity of this case. The patient's recent hip fracture and subsequent surgical repair, coupled with postoperative immobility and non-compliance with DVT prophylaxis, could significantly heighten her DVT risk. It is crucial to note that the combination of these factors, alongside evidence of long-term common iliac vein compression secondary to MTS, likely exacerbated the severity of thrombosis.

Research underscores the elevated risk of postoperative new-onset DVT (PNO-DVT) in patients following intertrochanteric fracture repair, particularly in those over 70 years old. Notably, the incidence of PNO-DVT is reported as 7.4%, with a majority of cases (82.1%) diagnosed within eight days post-surgery [9]. In contrast, this patient presented with extensive DVT more than 30 days after fracture repair and had only received enoxaparin for DVT prophylaxis for seven days post-operation.

This underscores the need for heightened vigilance regarding DVT risk in postoperative patients, especially those with additional predisposing factors such as MTS. Understanding the interplay of these risk factors is crucial for the optimal management and prevention of DVT complications in the future.

## Conclusions

In conclusion, this case underscores the importance of recognizing MTS as a significant factor contributing to DVT. DVT arises from a combination of genetic predispositions, both hereditary and acquired, as well as anatomical factors and lifestyle-related issues like immobility and trauma. Despite being frequently overlooked, anatomical abnormalities, such as MTS, play a crucial role in predisposing individuals to DVT. Addressing these anatomical factors, particularly through interventions like stenting may prove to be essential in preventing thrombotic events.

The case highlights the necessity of considering anatomical abnormalities like MTS to prevent DVT recurrence. This patient's experience of DVT post-fracture repair, worsened by non-compliance with prophylactic measures, stresses the importance of thorough thromboprophylaxis, especially for high-risk patients. Comprehensive evaluation and management of individual risk factors, including anatomical anomalies such as MTS, are crucial for effectively managing DVT-related complications.
